# Supplementary material for: Zika virus targets human trophoblast stem cells and prevents syncytialization in placental trophoblast organoids
Source: Nat Commun. 2023 Sep 8;14:5541. doi: 10.1038/s41467-023-41158-0 (PMC10491779; doi:10.1038/s41467-023-41158-0)
Supplement: Supplementary file 1 — Supplementary information [file 41467_2023_41158_MOESM1_ESM.pdf]

**Supplementary information**

**Supplementary figures**

**Table S1-8**

# Figure S1

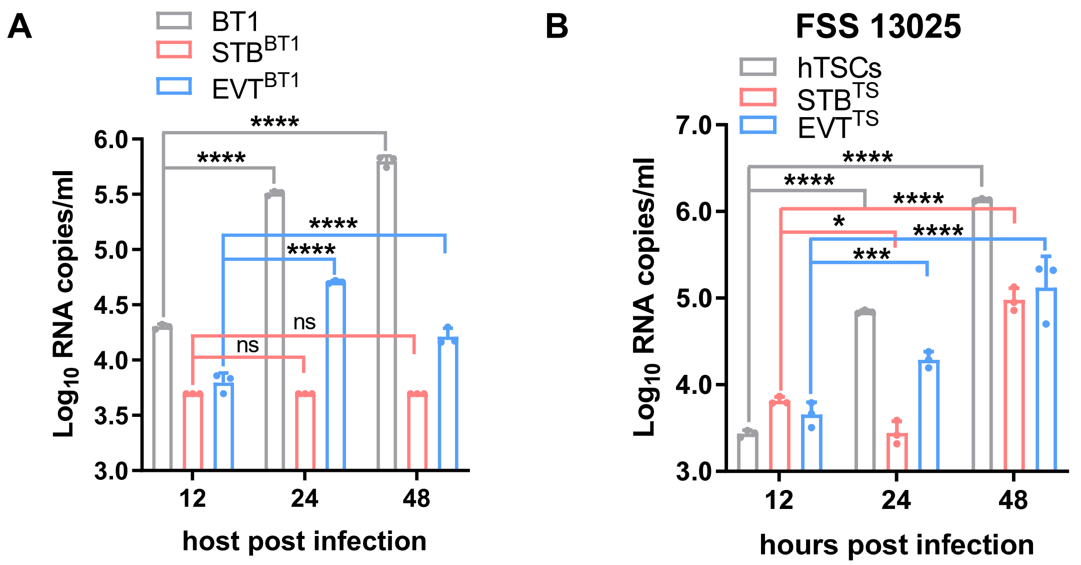


**Figure S1. Detection of ZIKV RNA in the supernatants of ZIKV-infected hTSC-derived trophoblast cells.**

(A) Quantification of ZIKV RNA in the supernatants of BT1, $STB^{BT1}$ and $EVT^{BT1}$ at 12, 24 and 48 hours post infection. The BT1, $STB^{BT1}$ and $EVT^{BT1}$ were infected with ZIKV at an MOI of 0.1. Two-way ANOVA analysis was used for statistical analysis of significance. n=3 independent experiments. ****, p<0.0001. ns, no significance.

(B) Quantification of ZIKV RNA in the supernatants of hTSCs, $STB^{TS}$ and $EVT^{TS}$ at 12, 24 and 48 hours post infection. The hTSCs, $STB^{TS}$ and $EVT^{TS}$ were infected with ZIKV strain FSS 13025 at an MOI of 0.1. Two-way ANOVA analysis was used for statistical analysis of significance. n=3 independent experiments. $STB^{TS}$, 12 hpi vs 24 hpi, *, p=0.18. $EVT^{TS}$, 12 hpi vs 24 hpi, ***, p=0.0002. ****, p<0.0001.

Data in this figure are shown as the mean ± s.d.

# Figure S2

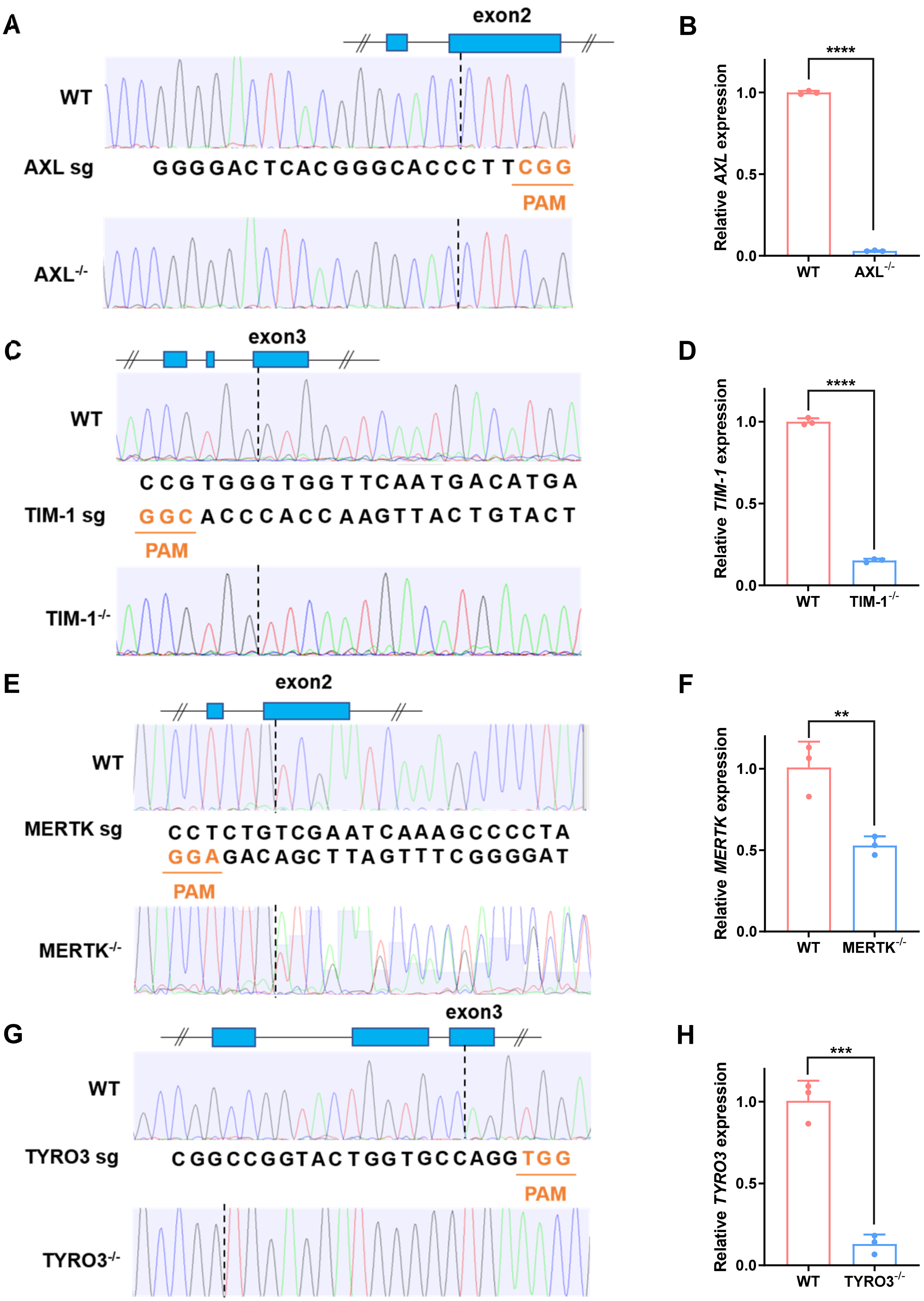


**Figure S2. Knockout of AXL, TIM-1, MERTK and TYRO3 in hTSCs using CRISPR/Cas9 system.**

(A, C, E, and G) Sanger sequencing results of sgRNA target sites in wildtype (WT), $AXL^{-/-}$ (panel A), $TIM\text{-}1^{-/-}$ (panel C), $MERTK^{-/-}$ (panel E) and $TYRO3^{-/-}$ (panel G) hTSCs. (B, D, F, and H) Quantification of AXL, TIM-1, TYRO3, and MERTK mRNA expression in WT, $AXL^{-/-}$ (panel B), $TIM\text{-}1^{-/-}$ (panel D), $MERTK^{-/-}$ (panel F) and $TYRO3^{-/-}$ (panel H) hTSCs. Two-tailed unpaired t test was used for statistical analysis of significance. n=3 independent experiments. Panel F, **, p=0.0079. Panel H, ***, p=0.0004. ****, p<0.0001. Data in this figure are shown as the mean ± s.d.

# Figure S3

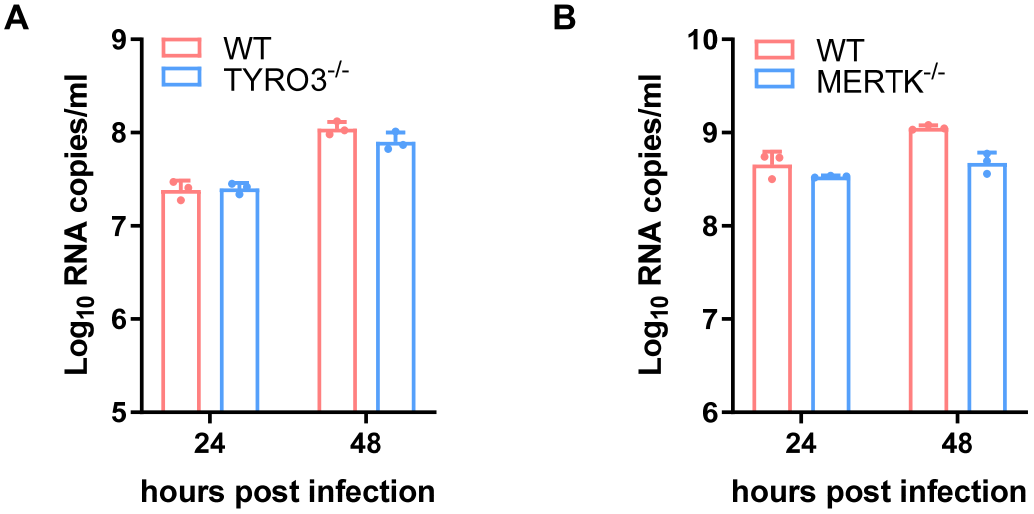


**Figure S3. Growth of ZIKV RNA in the supernatants of ZIKV-infected WT, $TYRO3^{-/-}$ and $MERTK^{-/-}$ hTSCs.**

(A and B) Quantification of ZIKV RNA in the supernatants of ZIKV-infected WT, $TYRO3^{-/-}$ (panel A) and $MERTK^{-/-}$ (panel B) hTSCs. The cells were exposed to ZIKV infection at an MOI of 0.1, and analyzed at 24 and 48 hours post infection. n=3 independent experiments. Data in this figure are shown as the mean ± s.d.

# Figure S4

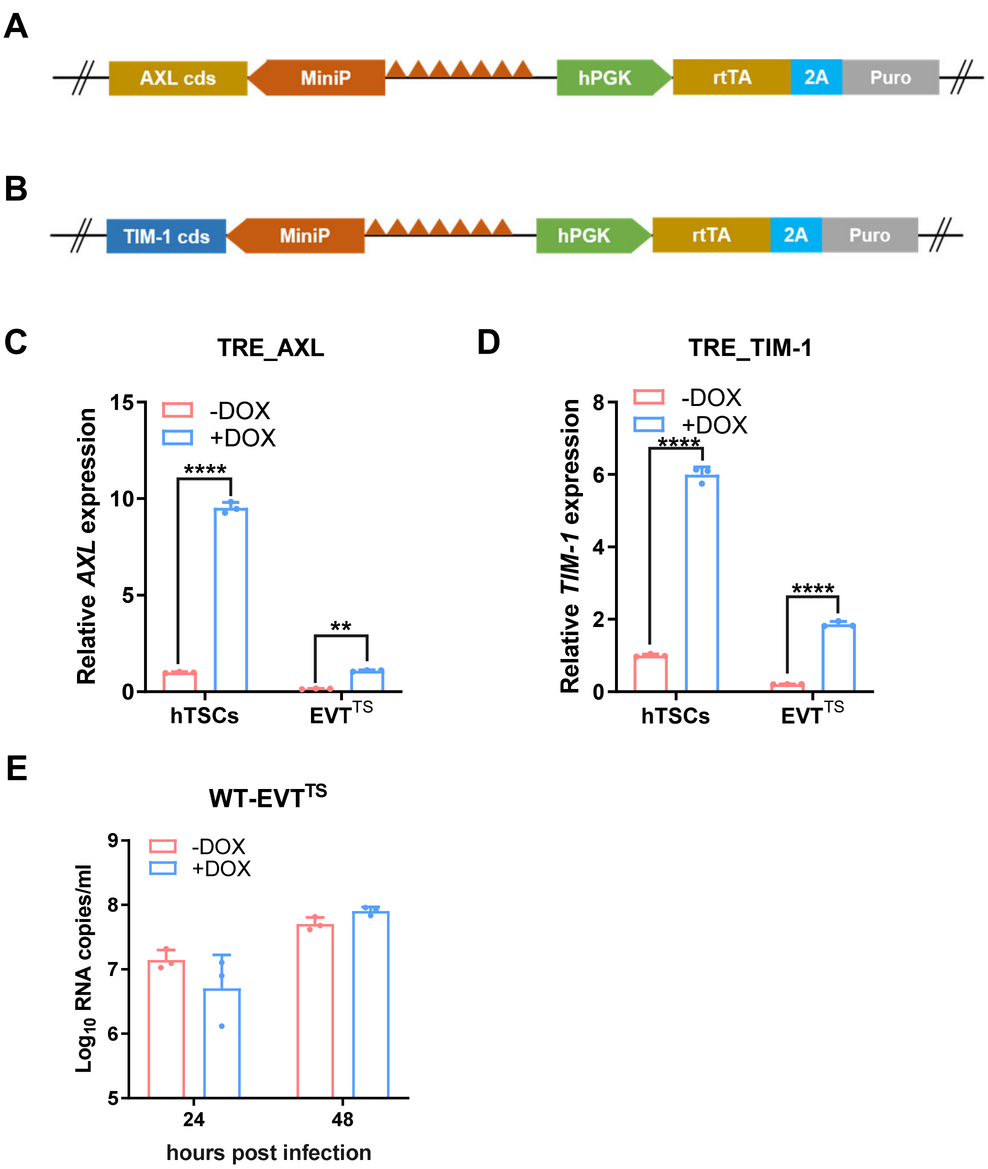


**Figure S4. Overexpression of AXL and TIM-1 in EVT$^{TS}$.**

(A and B) The lentiviral vectors used for DOX-induced overexpression of AXL (A) or TIM-1 (B) in EVT$^{TS}$. MiniP, mini promoter. ▲, tet operator. rtTA, a reverse transcriptional activator. 2A, P2A self-cleaving peptide. Puro, puromycin selection marker.

(C) Quantification of AXL expression in TRE_AXL-hTSCs and TRE_AXL-EVT$^{TS}$ under DOX induction. Two-way ANOVA analysis was used for statistical analysis of significance. n=3 independent experiments. **, p=0.0037. ****, p<0.0001.

(D) Quantification of TIM-1 expression in TRE_TIM-1-hTSCs and TRE_TIM-1-EVT$^{TS}$ under DOX induction. Two-way ANOVA analysis was used for statistical analysis of significance. n=3 independent experiments. ****, p<0.0001.

(E) Quantification of viral RNA in the supernatants of ZIKV-infected WT-EVT$^{TS}$ at 24 and 48 hours post infection. The WT-EVT$^{TS}$ was exposed to ZIKV at MOI 0.1. n=3 independent experiments.

Date in this figure are shown as the mean ± s.d.

# Figure S5

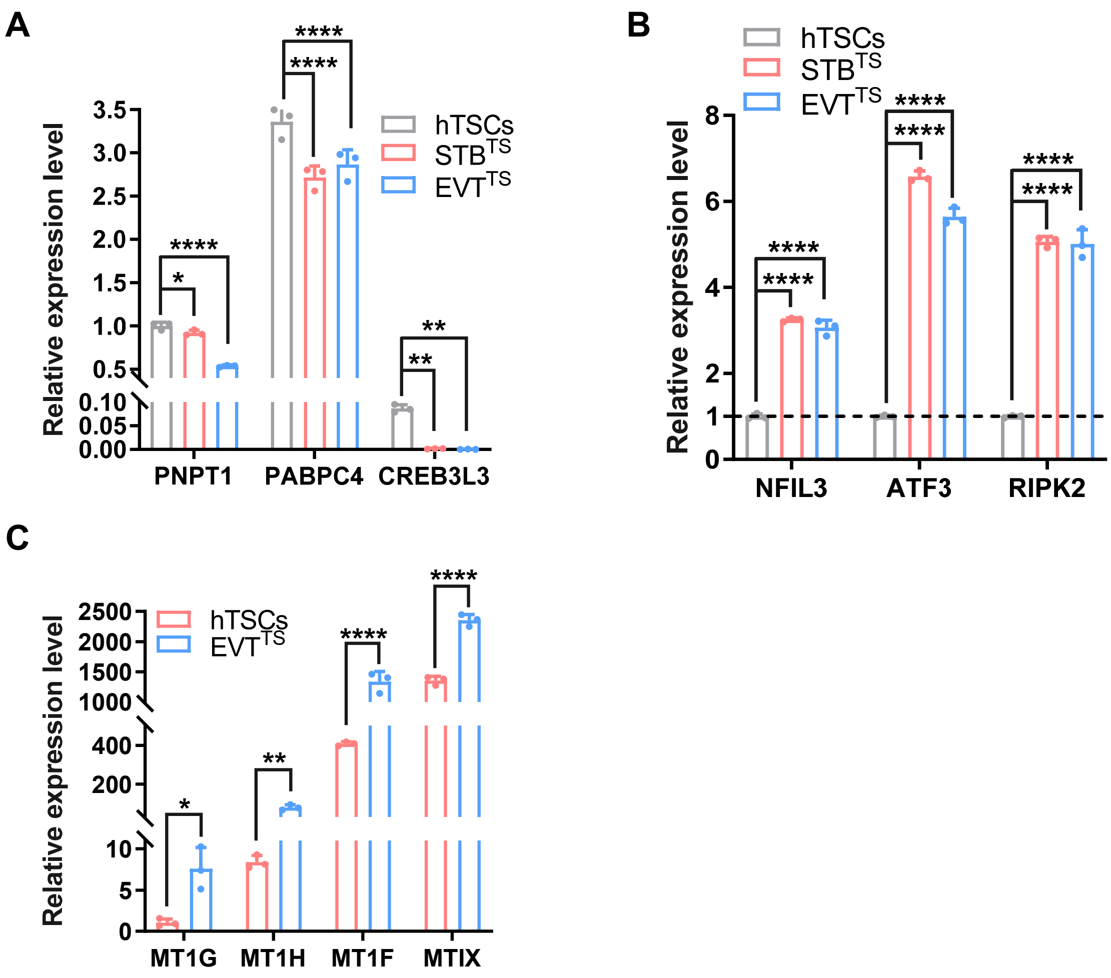


**Figure S5. qRT-PCR analysis of the ISG expression in hTSCs, $STB^{TS}$ and $EVT^{TS}$.**

(A) Quantification of PNPT1, PABPC4 and CREB3L3 mRNA expression in hTSCs, $STB^{TS}$ and $EVT^{TS}$. n=3 independent experiments. Two-way ANOVA analysis was used for statistical analysis of significance. PNPT1, hTSCs vs $STB^{TS}$, *, p=0.0114. CREB3L3, hTSCs vs $STB^{TS}$, **, p=0.0079. hTSCs vs $EVT^{TS}$, **, p=0.0072. ****, p<0.0001.

(B) Quantification of NFIL3, ATF3 and RIPK2 mRNA expression in hTSCs, $STB^{TS}$ and $EVT^{TS}$. n=3 independent experiments. The dashed line indicates the expression of the genes in hTSCs for normalization. Two-way ANOVA analysis was used for statistical analysis of significance. ****, p<0.0001.

(C) Quantification of MT1G, MT1H, MTIF and MT1X mRNA expression in hTSCs and $EVT^{TS}$. n=3 independent experiments. Two-way ANOVA analysis was used for statistical analysis of significance. *, p=0.0374. **, p=0.0066. ****, p<0.0001.

Data in this figure are shown as the mean ± s.d.

# Figure S6

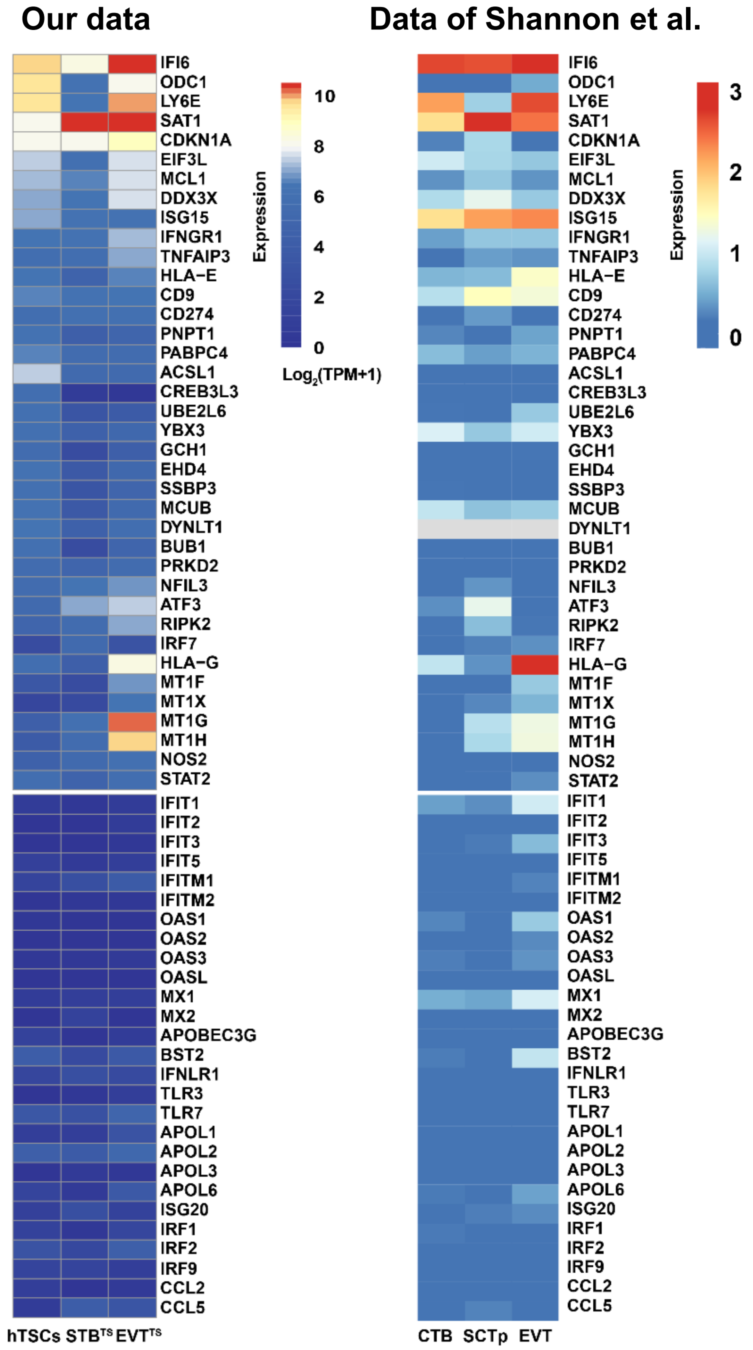


**Figure S6. The relative expression of ISGs in hTSCs/CTB, STB$^{TS}$/SCTp and EVT$^{TS}$/EVT of our and competing models.**

Heatmap showing the relative expression of ISGs with high (up) and low (bottom) expression in our hTSCs, STB$^{TS}$ and EVT$^{TS}$ (left panel), and in CTB, SCTp and EVT of competing models (right panel).

# Figure S7

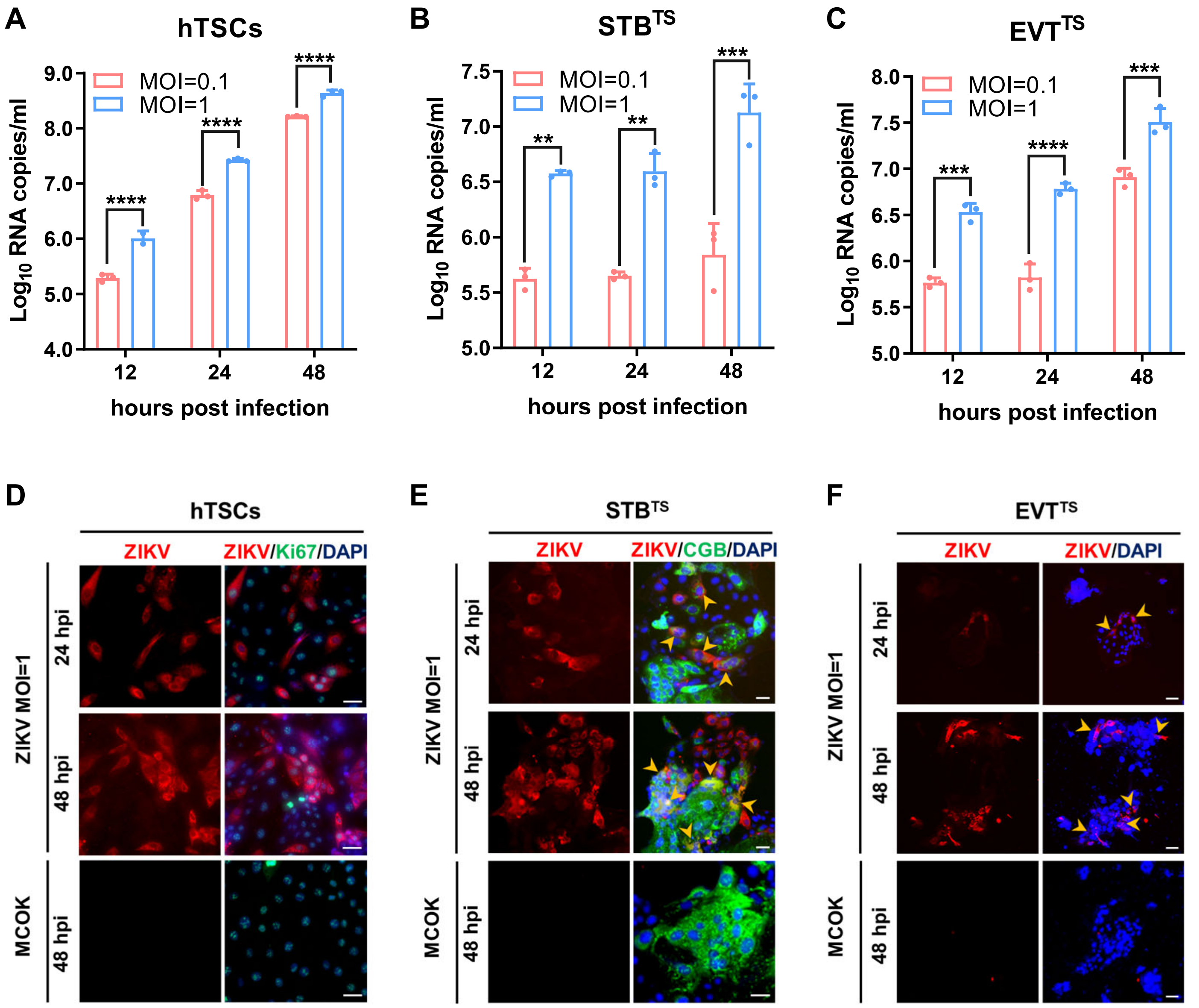


**Figure S7. Comparison of the ZIKV infection features of the hTSCs, $STB^{TS}$ and $EVT^{TS}$ infected with ZIKV at MOI 0.1 and 1.**

(A, B and C) Quantification of ZIKV RNA in the supernatants of hTSCs (panel A), $STB^{TS}$ (panel B) and $EVT^{TS}$ (panel C) at 12, 24 and 48 hours post infection. The hTSCs, $STB^{TS}$ and $EVT^{TS}$ were infected with at MOIs of 0.1 and 1. Two-way ANOVA analysis was used for statistical analysis of significance. n=3 independent experiments. Panel B, 12 hpi, MOI=0.1 vs MOI=1, **, p=0.0032. 24 hpi, MOI=0.1 vs MOI=1, **, p=0.0034. 48 hpi, MOI=0.1 vs MOI=1, ***, p=0.0006. Panel C, 12 hpi, MOI=0.1 vs MOI=1, ***, p=0.0001. 48 hpi. MOI=0.1 vs MOI=1, ***, p=0.0005. ****, p<0.0001.

(D, E and F) Immunofluorescence staining for Ki67 (a marker of proliferative CTB), CGB (a marker of STB) and ZIKV E protein in hTSCs (panel D), $STB^{TS}$ (panel E) and $EVT^{TS}$ (panel F). Nuclei were stained with DAPI. The hTSCs, $STB^{TS}$ and $EVT^{TS}$ were infected with ZIKV at an MOI of 1 and analyzed at 24 and 48 hours post infection. The yellow arrow heads indicated the positive intracellular ZIKV E signals in $STB^{TS}$ and $EVT^{TS}$. Scale bars: 50 μm.

Data in this figure are shown as the mean ± s.d.

# Figure S8

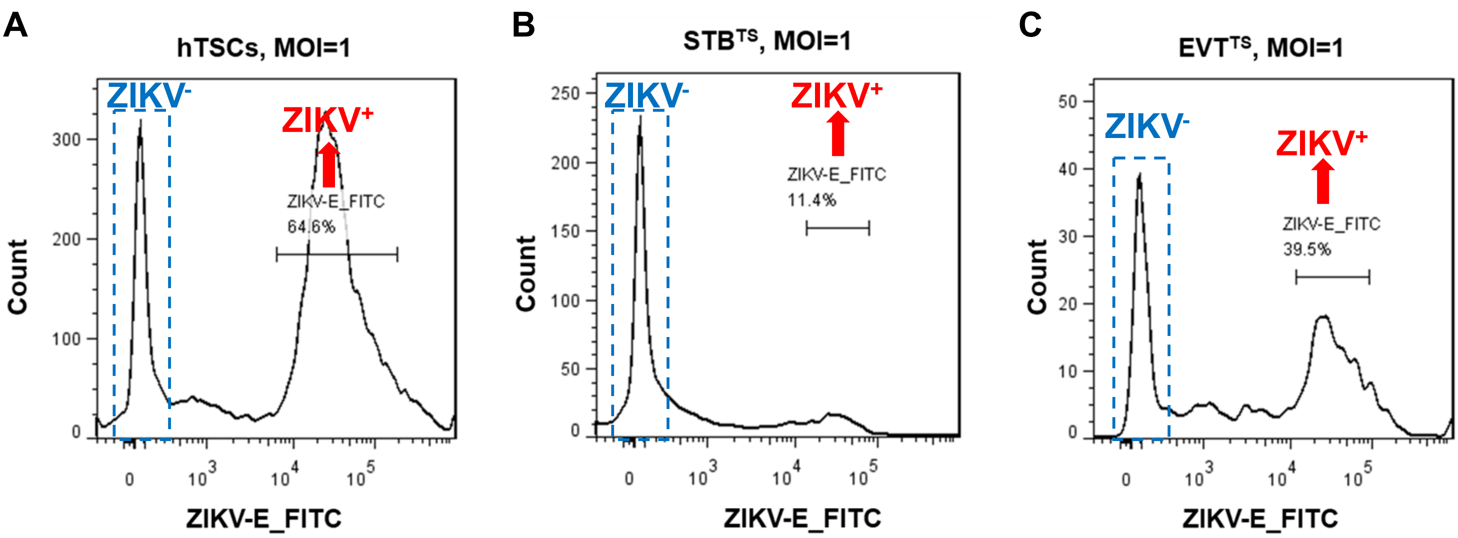


**Figure S8. FACS analysis of ZIKV-infected hTSCs, STB$^{TS}$ and EVT$^{TS}$.**
(A, B and C) FACS analysis of ZIKV-infected hTSCs (panel A), STB$^{TS}$ (panel B) and EVT$^{TS}$ (panel C) using ZIKV E protein antibody. The hTSCs, STB$^{TS}$ and EVT$^{TS}$ were infected with ZIKV at MOI 1 and analyzed at 48 hours post infection. Cells were stained for viral E protein and sorted into ZIKV-infected (ZIKV$^{+)}$ and uninfected (ZIKV$^{-}$) cells. Total RNA of ZIKV$^{+}$ and ZIKV$^{-}$ cells was extracted for qRT-PCR analysis.

# Figure S9

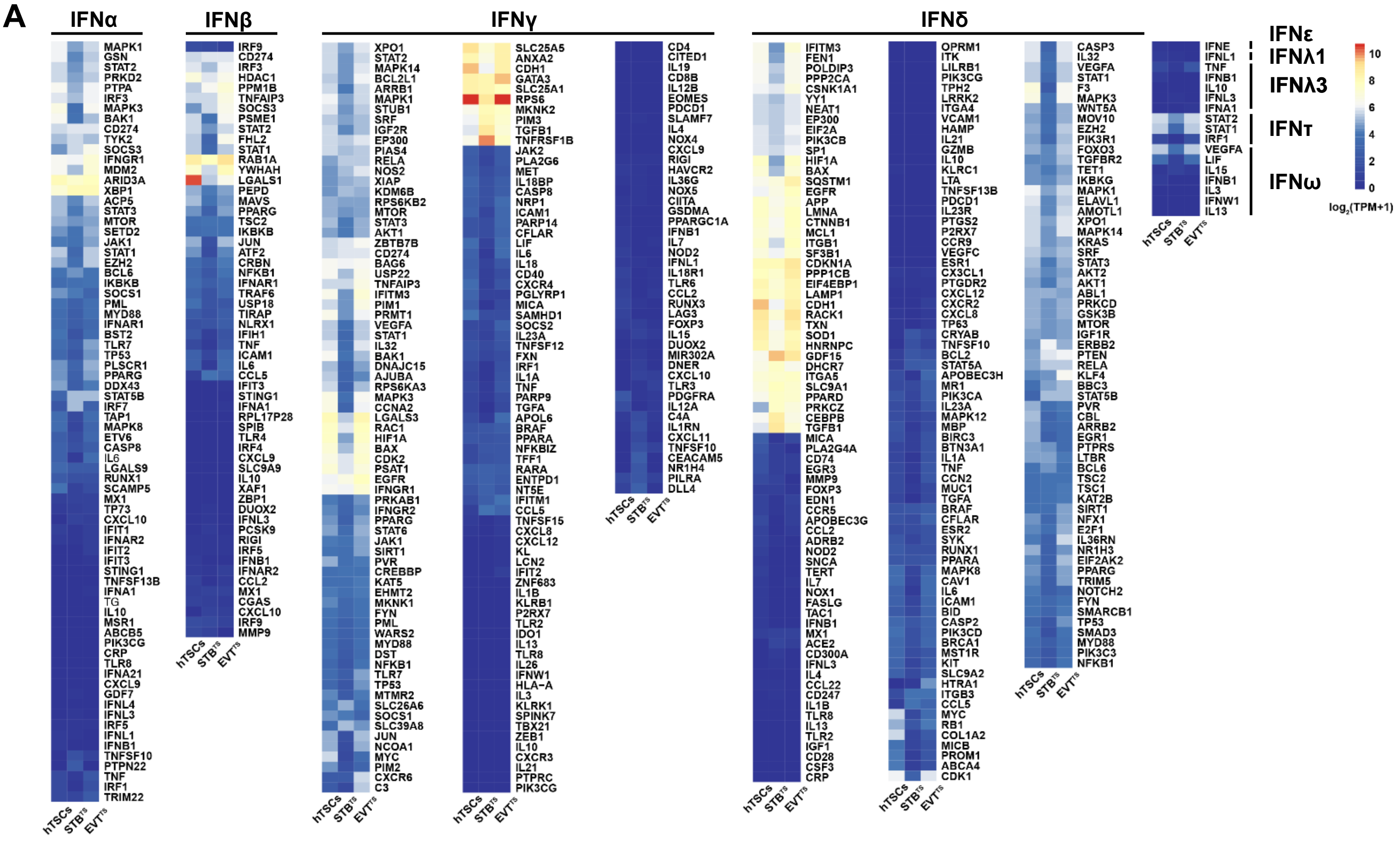


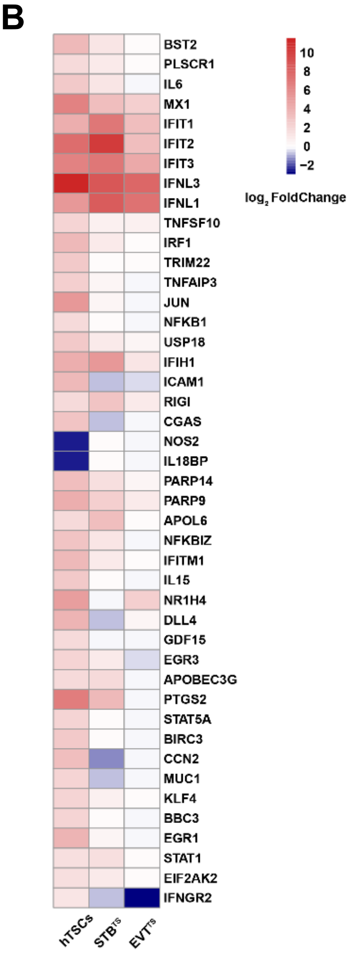


**Figure S9. The relative expression of IFNs in mock- and ZIKV-infected hTSCs, STB$^{TS}$ and EVT$^{TS}$.**

(A) Heatmap showing the z-acore TPM of IFNs in hTSCs, STB$^{TS}$ and EVT$^{TS}$.

(B) Heatmap showing changed IFNs in ZIKV-infected hTSCs, STB$^{TS}$ and EVT$^{TS}$.

# Figure S10

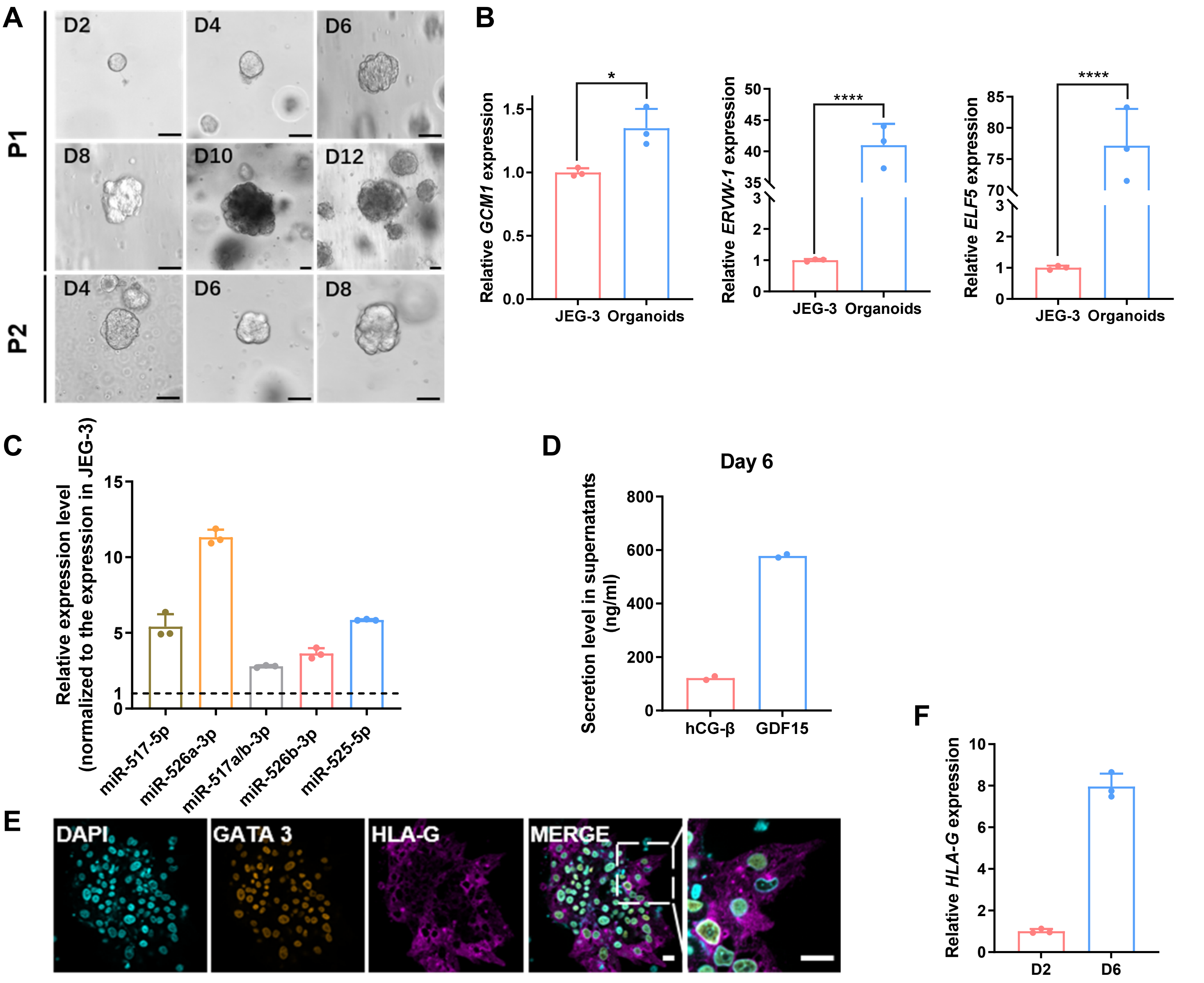


**Figure S10. Molecular characterizations of hTSC-organoids.**

(A) Brightfield images of passage 1 (P1) and passage 2 (P2) hTSC-organoids. D, Day. Scale bars: 50 μm.

(B) Quantification of GCM1, ERVW-1, and ELF5 mRNA expression in the choriocarcinoma line JEG-3 (positive controls) and hTSC-organoids. Two-tailed unpaired t test was used for statistical analysis of significance. n=3 independent experiments. Left panel, *, p=0.0177. ****, p<0.0001.

(C) Quantification of miRNAs miR-517-5p, miR-526a-3p, miR-517a/b-3p, miR-526b-3p, and miR-525-5p expression in the choriocarcinoma line JEG-3 (positive controls) and hTSC-derived organoids. Graph showing the relative expression levels of the miRNAs from in organoids to those of JEG-3. The dashed line indicates the expression of the genes in JEG-3 for normalization. n=3 independent experiments.

(D) Quantification of the hCG-β and GDF15 secreted by hTSC-organoids by ELISA. The amount of hCG-β and GDF15 secreted by organoids at day 6 was shown. n=2 independent experiments.

(E) Immunofluorescence staining for GATA 3 and HLA-G in the EVT cells derived from hTSC-organoids. Nuclei were stained with DAPI. Scale bars: 20 μm.

(F) Quantification of HLA-G mRNA expression in the hTSC-organoid-derived EVT at day 2 and day 6 after induction. n=3 independent experiments.

Data in panel B, C and F are shown as the mean ± s.d. Data in panel D are shown as mean.

# Figure S11

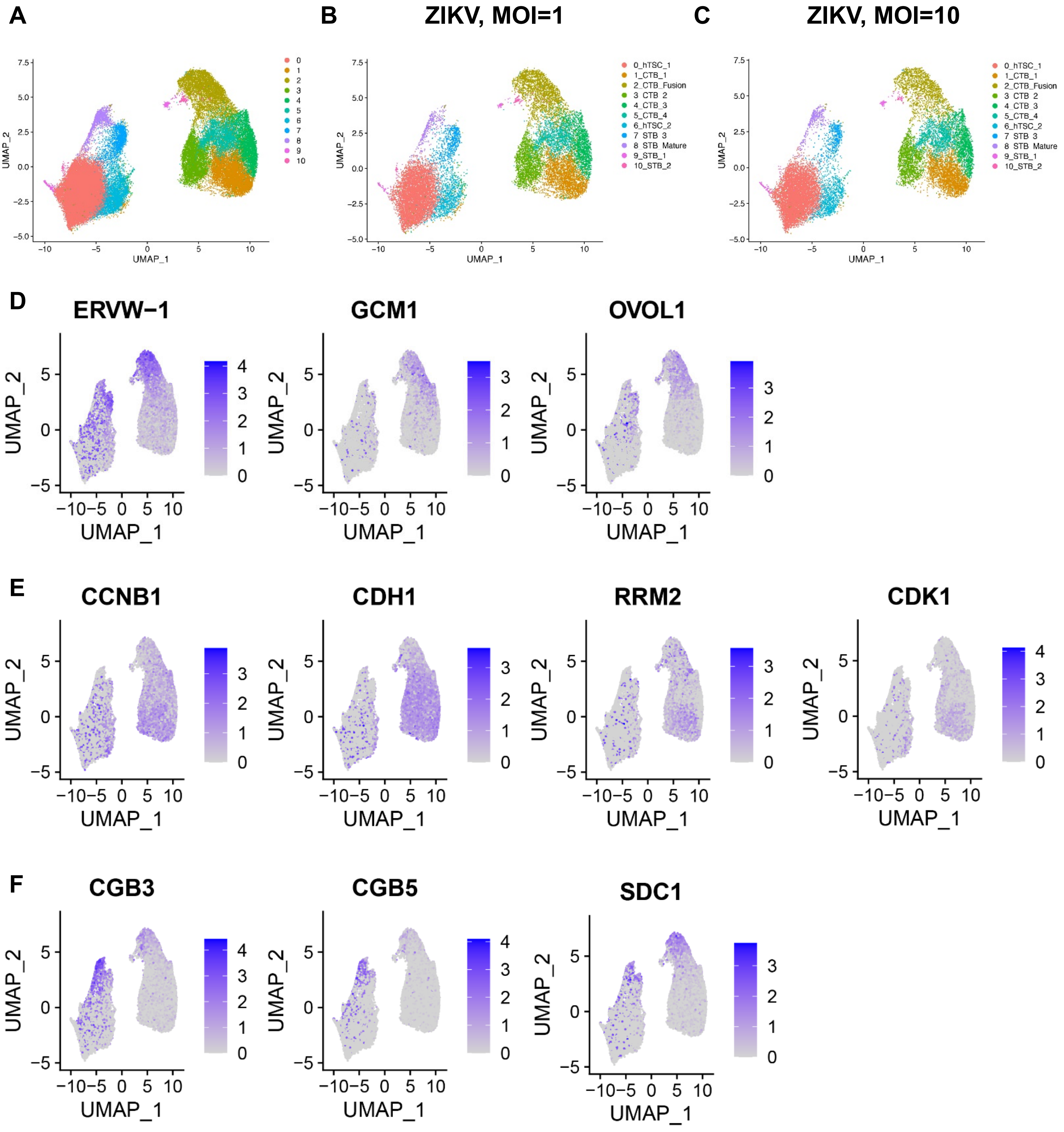


**Figure S11. The transcriptome profile of ZIKV-infected hTSC-organoids at single-cell resolution.**

(A) UMAP showing the integrated datasets of MOCK- and ZIKV-infected hTSC-organoids.

(B) UMAP showing the cell composition of ZIKV-infected hTSC-organoids. The hTSC-organoids were infected with ZIKV at MOIs of 1 (left panel) and 10 (right panel).

(D, E and F) UMAP showing the cells expressing CTB_Fusion (panel D), CTB (panel E) and STB (panel F) marker genes in hTSC-organoids.

# Figure S12

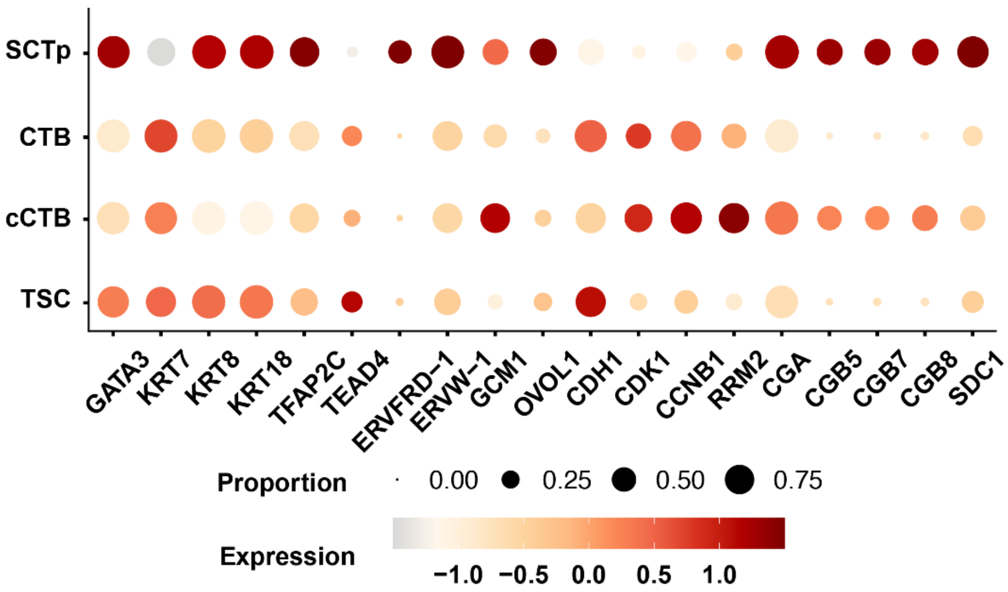


**Figure S12. The expression of trophoblast marker genes in the trophoblast cells of competing models.**

Dot plot indicating the expression of trophoblast marker genes in the scRNA-seq data of competing trophoblast organoids. SCTp, syncytiotrophoblast precursors. cCTB, column CTB-like cell. TSC, trophoblast stem cell.

# Figure S13

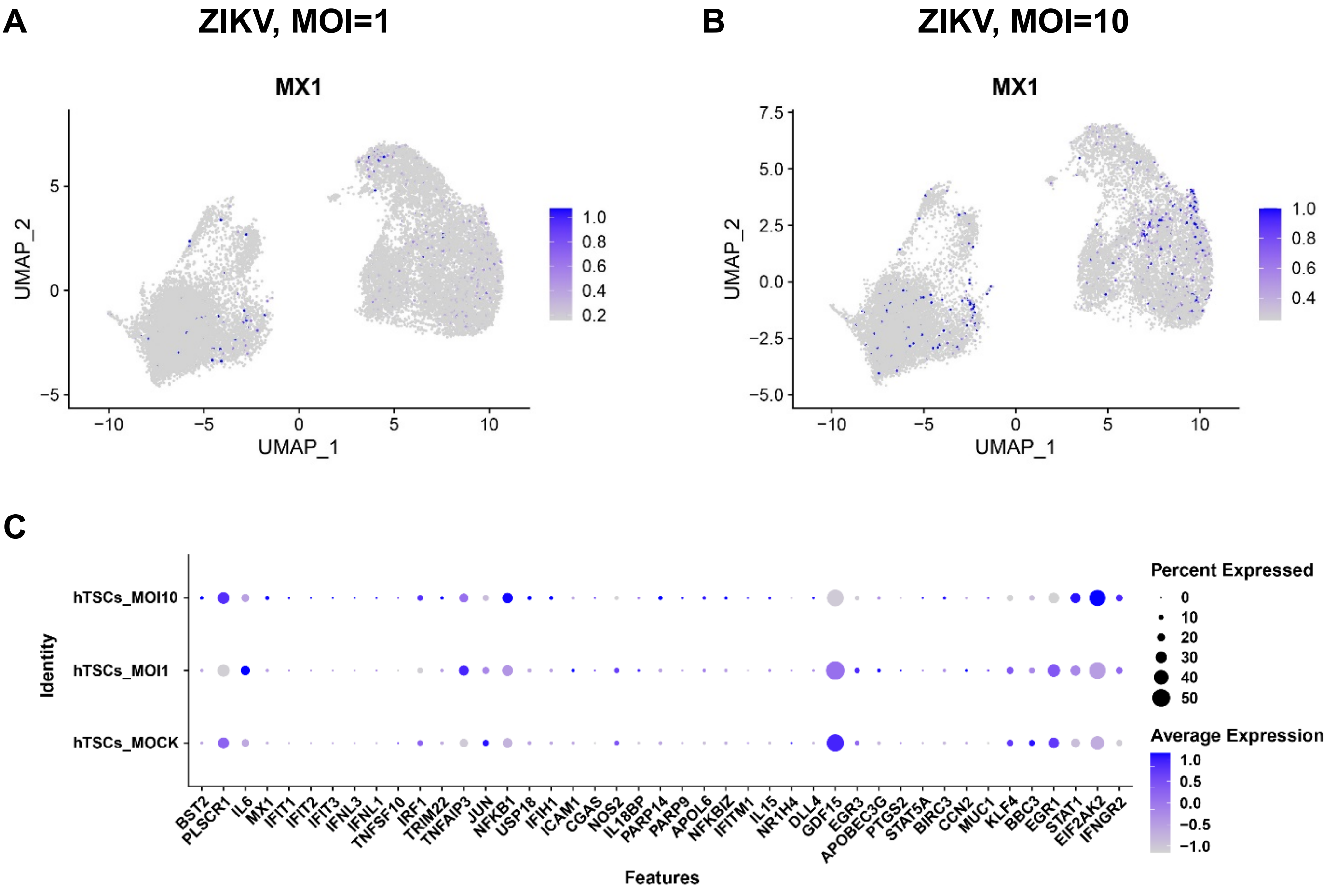


**Figure S13. The expression of IFNs in MX1-positive cells of ZIKV-infected hTSC-organoids.**

(A and B) UMAP showing the MX1-positive cells in the hTSC-organoids infected by ZIKV at an MOI of 1 (panel A) and 10 (panel B).

(C) Dot plot indicating the expression of the IFNs in the hTSCs from mock- and ZIKV (MOI 1 and 10)-infected hTSC-organoids.

# Figure S14

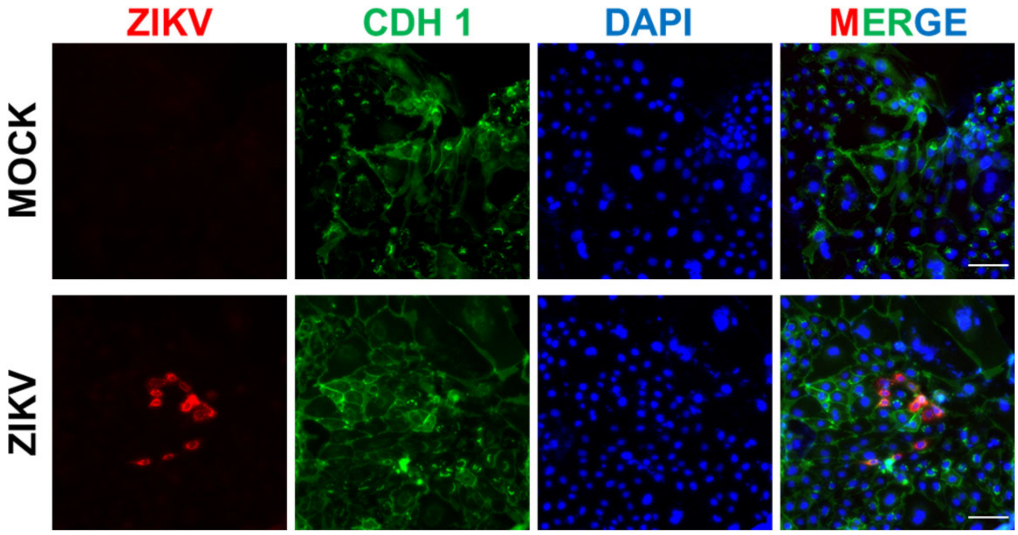


**Figure S14. Immunofluorescence staining for ZIKV and CDH 1 in STB$^{TS}$.**
Immunofluorescence staining for ZIKV and CDH 1 in STB$^{TS}$. The hTSCs were infected with ZIKV at an MOI of 1 and induced to STB$^{TS}$ and analyzed at 96 hours. The representative images were selected from three independent experiments. Nuclei were stained with DAPI. Scale bars: 100 µm.

# Figure S15

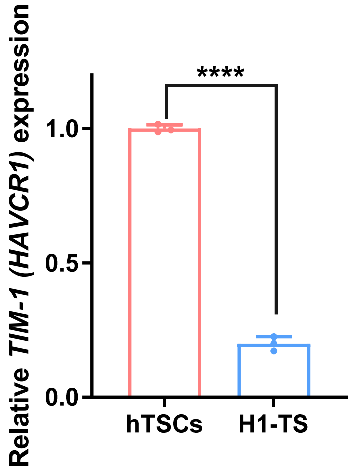


**Figure S15. The expression of TIM-1 (HAVCR1) in blastocyst-derived hTSCs and hESC-derived hTSC-like cells.**

Quantification of TIM-1 (HAVCR1) mRNA expression in blastocyst-derived hTSCs (hTSCs) and hESC-derived hTSC-like cells (H1-TS). Two-tailed unpaired t test was used for statistical analysis of significance. n=3 independent experiments. n=3 independent experiments. ****, $p<0.0001$. Data in this figure are shown as the mean ± s.d.

**Table S1. Statistical results of ZIKV-positive cells in the hTSCs and STB$^{TS}$ infected with ZIKV at 24 hpi.**

| | T | P | % |
|---|---|---|---|
| hTSCs | 84 | 9 | 10.71429 |
| | 134 | 6 | 4.477612 |
| | 91 | 3 | 3.296703 |
| | 27 | 6 | 22.22222 |
| | 76 | 7 | 9.210526 |
| | 93 | 7 | 7.526882 |
| | 154 | 6 | 3.896104 |
| | 49 | 5 | 10.20408 |
| | 68 | 6 | 8.823529 |
| | 58 | 6 | 10.34483 |
| STB$^{TS}$ | 65 | 3 | 4.615385 |
| | 86 | 5 | 5.813953 |
| | 103 | 3 | 2.912621 |
| | 140 | 6 | 4.285714 |

T, Total number of nuclei in the field; P, Number of ZIKA E-positive nuclei in the field; %, (P/T)×100.

**Table S2. Statistical results of ZIKV-positive cells in the hTSCs, $STB^{TS}$ and $EVT^{TS}$ infected with ZIKV at 48 hpi.**

| | T | P | % |
|---|---|---|---|
| hTSCs | 150 | 10 | 6.666667 |
| | 187 | 14 | 7.486631 |
| | 196 | 21 | 10.71429 |
| | 123 | 8 | 6.504065 |
| | 135 | 12 | 8.888889 |
| | 272 | 11 | 4.044118 |
| | 189 | 15 | 7.936508 |
| | 108 | 8 | 7.407407 |
| | 194 | 34 | 17.52577 |
| | 159 | 10 | 6.289308 |
| $STB^{TS}$ | 140 | 4 | 2.857143 |
| | 175 | 14 | 8 |
| | 172 | 10 | 5.813953 |
| | 101 | 6 | 5.940594 |
| | 93 | 3 | 3.225806 |
| | 111 | 15 | 13.51351 |
| | 172 | 8 | 4.651163 |
| | 102 | 7 | 6.862745 |
| $EVT^{TS}$ | 92 | 3 | 3.26087 |
| | 102 | 6 | 5.882353 |
| | 48 | 2 | 4.166667 |
| | 41 | 1 | 2.439024 |

T, Total number of nuclei in the field; P, Number of ZIKA E-positive nuclei in the field; %, $(P/T)\times100$.

**Table S3. Statistical results of the fusion index of the STB$^{TS}$ differentiated from ZIKV-infected hTSCs.**

| N | S | T |
|---|---|---|
| 80 | 9 | 303 |
| 63 | 7 | 319 |
| 54 | 10 | 280 |
| 97 | 9 | 283 |
| 31 | 9 | 371 |
| 58 | 6 | 488 |
| 72 | 7 | 311 |
| 47 | 10 | 382 |
| 42 | 8 | 304 |
| 46 | 8 | 246 |

N, Number of nuclei in syncytia; S, Number of syncytia; T, Total number of nuclei.

**Table S4. Statistical results of the fusion index of the STB$^{TS}$ differentiated from Mock-infected hTSCs.**

| N | S | T |
|---|---|---|
| 79 | 12 | 230 |
| 61 | 10 | 281 |
| 82 | 11 | 253 |
| 115 | 9 | 221 |
| 32 | 8 | 232 |
| 59 | 7 | 212 |
| 72 | 8 | 209 |
| 59 | 10 | 198 |
| 62 | 10 | 272 |
| 75 | 9 | 225 |

N, Number of nuclei in syncytia; S, Number of syncytia; T, Total number of nuclei.

## Table S5. Primers for qPCR and qRT-PCR.

| Primer | Sequence (5'-3') |
|---|---|
| ZIKV-F | GGTCAGCGTCCTCTCTAATAAACG |
| ZIKV-R | GCACCCTAGTGTCCACTTTTTCC |
| ZIKV-Probe | FAM-AGCCATGACCGACACCACACCGT-BHQ1 |
| AXL-RT-F | CCGTGGACCTACTCTGGCT |
| AXL-RT-R | CCTTGGCGTTATGGGCTTC |
| TIM-1-RT-F | TGGCAGATTCTGTAGCTGGTT |
| TIM-1-RT-R | AGAGAACATGAGCCTCTATTCCA |
| TYRO3-RT-F | CAGCCGGTGAAGCTCAACT |
| TYRO3-RT-R | TGGCACACCTTCTACCGTGA |
| MERTK-RT-F | CTCTGGCGTAGAGCTATCACT |
| MERTK-RT-R | AGGCTGGGTTGGTGAAAACA |
| GCM1-RT-F | TTCCCGGTCACCAACTTCTG |
| GCM1-RT-R | GTAAACTCCCCTGACTTTGTGTT |
| ERVW-1-RT-F | ATGCCCCGCAACTGCTATC |
| ERVW-1-RT-R | AGACAGTGACTCCAAGTCCTC |
| ERVFRD-1-RT-F | ACCGCCATCCTGATTTCCC |
| ERVFRD-1-RT-R | GAGGCTGGATAAGCTGTCCC |
| OVOL1-RT-F | TGAACATGAGCCTTCGAGACT |
| OVOL1-RT-R | CAAGGGTCACCTTCATCTTGG |
| HLA-G-RT-F | GAGGAGACACGGAACACCAAG |
| HLF-G-RT-R | GTCGCAGCCAATCATCCACT |
| miR-517-5p-RT-F | TCGGCAGGCCTCTAGATGGAAG |
| miR-526a-3p-RT-F | TCGGCAGGGAAAGCGCTTCCTT |
| miR-517a/b-3p-RT-F | TCGGCAGGATCGTGCATCCCTT |
| miR-526b-3p-RT-F | TCGGCAGGGAAAGTGCTTCCTT |
| miR-525-5p-RT-F | TCGGCAGGGAAGGCGCTTCCCT |

RT, Real-time; F, Forward; R, Reverse.

## Table S6. sgRNAs for gene knockout.

| Primer | Sequence (5'-3') |
|---|---|
| AXL sgRNA-F | GGGGACTCACGGGCACCCTT |
| AXL sgRNA-R | AAGGGTGCCCGTGAGTCCCC |
| TIM-1 sgRNA-F | TCATGTCATTGAACCACCCA |
| TIM-1 sgRNA-R | TGGGTGGTTCAATGACATGA |
| TYRO3 sgRNA-F | CGGCCGGTACTGGTGCCAGG |
| TYRO3 sgRNA-R | CCTGGCACCAGTACCGGCCG |
| MERTK sgRNA-F | TAGGGGCTTTGATTCGACAG |
| MERTK sgRNA-R | CTGTCGAATCAAAGCCCCTA |

F, Forward; R, Reverse.

## Table S7. Primers for genotyping.

| Primer | Sequence (5'-3') |
|---|---|
| AXL-GT-F | AGCGCGACCTGTTAAGTCTC |
| AXL-GT-R | AGTGGTCAAACTGGGGTTCC |
| TIM-1-GT-F | CCACTACAGTGGAGCTGTCA |
| TIM-1-GT-R | AGGACTTACGGGAACCTCCTC |
| TYRO3-GT-F | AGTTGTACATCCCAGTCAGCG |
| TYRO3-GT-R | GCTCAAGCAACGGAAACAGA |
| MERTK-GT-F | CTTGAGCCCTGCTGTGTCAT |
| MERTK-GT-R | TGATGTGCCCCAAGCAATTC |

GT, Genotyping; F, Forward; R, Reverse.

**Table S8. qRT-PCR primers for detection of ISG expression**

| Primer (F, Forward; R, Reverse) | Sequence (5'-3') |
|---|---|
| IFI6-RT-F | GGTCTGCGATCCTGAATGGG |
| IFI6-RT-R | TCACTATCGAGATACTTGTGGGT |
| ODC1-RT-F | TTTACTGCCAAGGACATTCTGG |
| ODC1-RT-R | GGAGAGCTTTTAACCACCTCAG |
| LY6E-RT-F | CAGCTCGCTGATGTGCTTCT |
| LY6E-RT-R | CAGACACAGTCACGCAGTAGT |
| SAT1-RT-F | ACCCGTGGATTGGCAAGTTAT |
| SAT1-RT-R | TGCAACCTGGCTTAGATTCTTC |
| CDKN1A-RT-F | TGTCCGTCAGAACCCATGC |
| CDKN1A-RT-R | AAAGTCGAAGTTCCATCGCTC |
| EIF3L-RT-F | TGACCCCTACGCTTATCCCAG |
| EIF3L-RT-R | GTTTGCTGTTCATACTGACGTTC |
| MCL1-RT-F | TGCTTCGGAAACTGGACATCA |
| MCL1-RT-R | TAGCCACAAAGGCACCAAAAG |
| DDX3X-RT-F | AGCAGTTTTGGATCTCGTAGTG |
| DDX3X-RT-R | ACTGTTTCCACCACGTTCAAAT |
| ISG15-RT-F | CGCAGATCACCCAGAAGATCG |
| ISG15-RT-R | TTCGTCGCATTTGTCCACCA |
| IFNGR1-RT-F | TCTTTGGGTCAGAGTTAAAGCCA |
| IFNGR1-RT-R | TTCCATCTCGGCATACAGCAA |
| TNFAIP3-RT-F | TCCTCAGGCTTTGTATTTGAGC |
| TNFAIP3-RT-R | TGTGTATCGGTGCATGGTTTTA |
| IFIT1-RT-F | AGAAGCAGGCAATCACAGAAAA |
| IFIT1-RT-R | CTGAAACCGACCATAGTGGAAAT |
| IFIT2-RT-F | AAGCACCTCAAAGGGCAAAAC |
| IFIT2-RT-R | TCGGCCCATGTGATAGTAGAC |
| IFIT3-RT-F | AGAAAAGGTGACCTAGACAAAGC |
| IFIT3-RT-R | CCTTGTAGCAGCACCCAATCT |
| IFIT5-RT-F | GGCCAAAATAAAGACGCCCTT |
| IFIT5-RT-R | GACCAGGCTTCGTACTTCTTC |
| IFITM1-RT-F | CCAAGGTCCACCGTGATTAAC |
| IFITM1-RT-R | ACCAGTTCAAGAAGAGGGTGTT |
| IFITM2-RT-F | ATGAACCACATTGTGCAAACCT |
| IFITM2-RT-R | CCCAGCATAGCCACTTCCT |
| TLR3-RT-F | TTGCCTTGTATCTACTTTTGGGG |
| TLR3-RT-R | TCAACACTGTTATGTTTGTGGGT |
| TLR7-RT-F | TCCTTGGGGCTAGATGGTTTC |
| TLR7-RT-R | TCCACGATCACATGGTTCTTTG |
| IRF1-RT-F | ATGCCCATCACTCGGATGC |
| IRF1-RT-R | CCCTGCTTTGTATCGGCCTG |
| IRF2-RT-F | CATGCGGCTAGACATGGGTG |
| IRF2-RT-R | GCTTTCCTGTATGGATTGCCC |
| IRF7-RT-F | GCTGGACGTGACCATCATGTA |
| IRF7-RT-R | GGGCCGTATAGGAACGTGC |
| IRF9-RT-F | GCCCTACAAGGTGTATCAGTTG |
| IRF9-RT-R | TGCTGTCGCTTTGATGGTACT |
| IFIH1-RT-F | TCGAATGGGTATTCCACAGACG |
| IFIH1-RT-R | GTGGCGACTGTCCTCTGAA |
| MX1-RT-F | GTTTCCGAAGTGGACATCGCA |
| MX1-RT-R | CTGCACAGGTTGTTCTCAGC |
| EPSTI1-RT-F | ACCCGCAATAGAGTGGTGAAC |
| EPSTI1-RT-R | GCTATCAAGGTGTATGCACTTGT |
| OAS1-RT-F | TGTCCAAGGTGGTAAAGGGTG |
| OAS1-RT-R | CCGGCGATTTAACTGATCCTG |
| OAS2-RT-F | CTCAGAAGCTGGGTTGGTTTAT |
| OAS2-RT-R | ACCATCTCGTCGATCAGTGTC |
| OAS3-RT-F | GAAGGAGTTCGTAGAGAAGGCG |

| | |
|---|---|
| OAS3-RT-R | CCCTTGACAGTTTTCAGCACC |
| OASL-RT-F | CTGATGCAGGAACTGTATAGCAC |
| OASL-RT-R | CACAGCGTCTAGCACCTCTT |
| APOL1-RT-F | TGGACTACGGAAAGAAGTGGT |
| APOL1-RT-R | CCTCCTTCAATTTGTCAAGGCTT |
| APOL6-RT-F | ACCAGGCGGAGAGAGAAAGT |
| APOL6-RT-R | TGTAGCTCCACGTCTTCACAC |
| IFI27-RT-F | TGCTCTCACCTCATCAGCAGT |
| IFI27-RT-R | CACAACTCCTCCAATCACAACT |
| PARP12-RT-F | GCTTGACAACCGAACACAACC |
| PARP12-RT-R | GGCATAGCTCATTATAGCTCAGG |
| EIF2AK2-RT-F | GCCGCTAAACTTGCATATCTTCA |
| EIF2AK2-RT-R | TCACACGTAGTAGCAAAAGAACC |
| IFITM3-RT-F | TGTCCAAACCTTCTTCTCTCC |
| IFITM3-RT-R | CGTCGCCAACCATCTTCC |
| PNPT1-RT-F | GCGAGCACTATGGAGTAGCG |
| PNPT1-RT-R | GCAGTGTCACCTGACTGTACTA |
| PABPC4-RT-F | AAGCCAATCCGCATCATGTG |
| PABPC4-RT-R | CTCTTGGGTCTCGAAGTGGAC |
| CREB3L3-RT-F | ATGAATACGGATTTAGCTGCTGG |
| CREB3L3-RT-R | AGGAAGTCGTCAGAGTCGGG |
| MT1G-RT-F | CTTCTCGCTTGGGAACTCTA |
| MT1G-RT-R | AGGGGTCAAGATTGTAGCAAA |
| MT1H-RT-F | ATCTGCAAAGGGGCGTCAG |
| MT1H-RT-R | GAATGTAGCAAATGAGTCGGAGTT |
| MT1F-RT-F | GAATGTAGCAAATGGGTCAAGGTG |
| MT1F-RT-R | TCTCCTGCACCTGCGCTGGT |
| MT1X-RT-F | GACCCCAACTGCTCCTGCTCG |
| MT1X-RT-R | GATGTAGCAAACGGGTCAGGGTTGTAC |
